# Role of Cardiovascular Magnetic Resonance in Native Valvular Regurgitation: A Comprehensive Review of Protocols, Grading of Severity, and Prediction of Valve Surgery

**DOI:** 10.3389/fcvm.2022.881141

**Published:** 2022-07-07

**Authors:** Emmanuelle Vermes, Laura Iacuzio, Franck Levy, Yohann Bohbot, Cédric Renard, Bernhard Gerber, Sylvestre Maréchaux, Christophe Tribouilloy

**Affiliations:** ^1^Department of Cardiology, Amiens University Hospital, Amiens, France; ^2^Department of Cardiology, Center Cardio-Thoracique de Monaco, Monaco, Monaco; ^3^UR UPJV 7517, Jules Verne University of Picardie, Amiens, France; ^4^Department of Radiology, Amiens University Hospital, Amiens, France; ^5^Division of Cardiology, Department of Cardiovascular Diseases, Cliniques Universitaires St. Luc, Pôle de Recherche Cardiovasculaire (CARD), Institut de Recherche Expérimentale et Clinique (IREC), Université catholique de Louvain, Brussels, Belgium; ^6^Department of Cardiology, Heart Valve Center, Lille Catholic University Hospital, Lille, France

**Keywords:** valvular regurgitation, cardiovascular magnetic resonance, echocardiography, regurgitant volume, regurgitant fraction

## Abstract

Valvular regurgitation is common in developed countries with an increasing prevalence due to the aging of the population and more accurate diagnostic imaging methods. Echocardiography is the gold standard method for the assessment of the severity of valvular heart regurgitation. Nonetheless, cardiovascular magnetic resonance (CMR) has emerged as an additional tool for assessing mainly the severity of aortic and mitral valve regurgitation in the setting of indeterminate findings by echocardiography. Moreover, CMR is a valuable imaging modality to assess ventricular volume and flow, which are useful in the calculation of regurgitant volume and regurgitant fraction of mitral valve regurgitation, aortic valve regurgitation, tricuspid valve regurgitation, and pulmonary valve regurgitation. Notwithstanding this, reference values and optimal thresholds to determine the severity and prognosis of valvular heart regurgitation have been studied lesser by CMR than by echocardiography. Hence, further larger studies are warranted to validate the potential prognostic relevance of the severity of valvular heart regurgitation determined by CMR. The present review describes, analyzes, and discusses the use of CMR to determine the severity of valvular heart regurgitation in clinical practice.

## Introduction

The prevalence of valvular regurgitation is increasing worldwide, especially in high-income countries (1, 2). In the setting of either atrioventricular or ventricular–arterial valve regurgitation, a large regurgitant volume is accommodated by chamber dilation of the ventricles to preserve compliance and forward cardiac output. However, after some time, ventricular remodeling and afterload mismatch eventually progress to a stage in which wall stress can no longer be maintained and preload reserve is overwhelmed, leading to heart failure. Thus, patients undergo progression from a compensated stage to subclinical ventricular dysfunction prior to decompensation and irreversible myocardial damage. These hemodynamic and pathological responses take place insidiously so that patients often remain asymptomatic for a long duration despite the occurrence of adverse ventricular remodeling. This makes the optimal timing of intervention in the setting of most valvular regurgitations difficult.

Current guidelines are mostly symptom-based and recommend intervention for severe valvular regurgitation in symptomatic patients or the presence of certain high-risk events (3, 4). The two main objectives of clinicians are, therefore, to accurately diagnose severe valvular regurgitation and identify high-risk features of early adverse ventricular remodeling in asymptomatic patients.

Echocardiography is the most practical diagnostic method for the assessment of valvular pathology and the current first-line imaging modality for this purpose due to the excellent visualization of valve anatomy it affords, its availability, and its ease of use. This cardiovascular imaging technique allows the user to analyze valve morphology and motion and the valvular annulus, quantify valvular regurgitation using different methods, and assess ventricle size and function (5). The EACVI/ASE guidelines propose a multiparametric approach for the evaluation of valvular regurgitation severity with the quantitative estimation of effective regurgitant orifice and regurgitant volume, the preferred technique when available (6). However, echocardiography assessment of valvular regurgitation suffers from several pitfalls, including a poor acoustic window for certain patients and specific issues concerning the vena contracta and flow convergence methods, especially in the presence of eccentric jets, non-circular regurgitant orifices, constrained PISA, variable jet intensity during the cardiac cycle, and multiple regurgitant jets (7, 8). The evaluation of regurgitation of the right heart and particularly the pulmonary valve is even more difficult by echocardiography.

Over the last 20–25 years, cardiovascular magnetic resonance (CMR) has emerged not only as the gold standard method for assessing left and right ventricular volume, mass, and function but also as a robust and accurate tool for evaluating volumetric quantification and accurate flow using a number of methods independent of jet morphology. However, recent guidelines still consider CMR as a “second tool” when echocardiographic parameters are inconsistent due to the absence of large prospective studies defining the clinical impact of valvular regurgitation quantification by CMR and due to its availability, time, and cost. It is therefore essential to define, in light of the most recent available data, the crucial role of this modality, which can provide, in an “all-in-one technique,” a unique approach to study both valve lesions and their consequences on the heart chambers and hemodynamics. In this review, we discuss the emerging potential of CMR for the diagnosis and prognosis of regurgitant lesions. We will detail for each type of valvular regurgitation (mitral, aortic, tricuspid, and pulmonary) its ability to assess regurgitation severity, the consequences for the ventricles, and we will propose a CMR-specific cut-off to help in the decision-making process for valve replacement/repair.

## Mitral Regurgitation

Moderate or severe mitral regurgitation (MR) is frequent in the general population and represents an important cause of morbidity and mortality worldwide (9). In the United States, its prevalence in patients ≥ 75 years has risen to > 4% (10) and almost to 8% in the United Kingdom (11).

Cardiac imaging is critical for evaluating the cause of MR (primary or secondary), assessing its severity and the consequences for the LV, and defining the best surgical timing.

### Cardiovascular Magnetic Resonance for Assessing the Cause of Mitral Regurgitation

#### 2D Cine Imaging for Valve Morphology

Assessing the cause of MR requires visualization of the anterior and posterior mitral valve (MV) leaflets, the mitral annulus, the chordae, the anterolateral and posterolateromedial papillary muscle, and left ventricular (LV) wall motion abnormalities. Although echocardiography (transthoracic and especially 3D transesophageal) is an excellent imaging of the MV which CMR cannot match, CMR can help in identifying MV morphology and MR mechanisms. With a high signal-to-noise ratio and an excellent blood–myocardium contrast-to-noise ratio, cardiac balanced steady-state free precession (bSSFP) imaging is the method of choice for cine (motion) images. Retrospective cardiac gating, corresponding to the continuous acquisition of both ECG and image signals collected over several consecutive heartbeats and used after image reconstruction, should be used to allow optimized temporal and spatial resolution. In the setting of atrial fibrillation, however, prospective gating may result in better image quality. According to recent SCMR recommendations and a consensus on the use of CMR in MR (12, 13), the morphology and motion of the MV apparatus can be studied using

**FIGURE 1:**
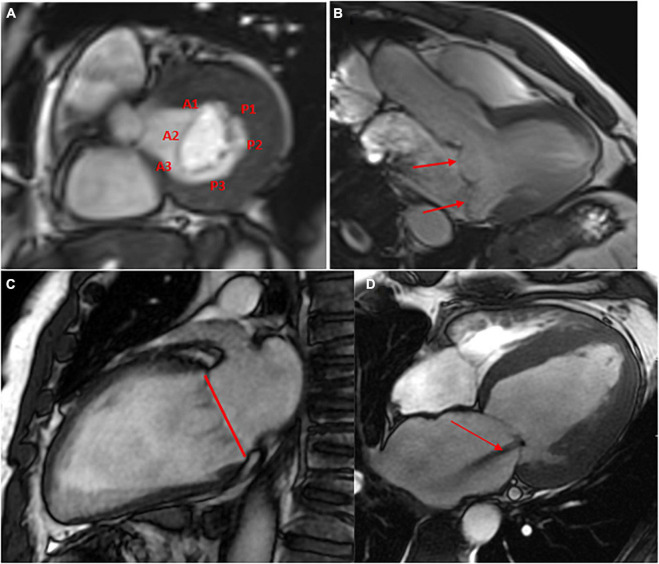
Anterior and posterior mitral valves and their scallops (A1, A2 and A3 and P1, P2, P3) visualized in diastole using balanced steady-state free precession (bSSFP) cine images in the plane of the mitral valve **(A)**. Cine 4 chamber view in a patient with posterior and anterior mitral valve prolapse (red arrows) **(B)**. Example of mitral annular measurement from the anterior commissure to the posterior commissure (red line) in the 2-chamber view **(C)**. A central mitral regurgitation (flow void in the left atrium, red arrow) secondary to mitral annular dilatation visualized in the 4-chamber view **(D)**.

- A stack of contiguous thin (slice thickness between 5 and 7 mm) bSSFP cines in the short axis covering the MV.
- A stack of contiguous cines through-plane on the MV and perpendicular to the mitral commissure to cover all of the mitral scallop; from A1–P1 to A2–P2 and A3–P3 (Figure 1).
- Standard long-axis cine images, including a 2-chamber view (vertical), a 3-chamber view [left ventricular outflow tract (LVOT)-1 view], and a 4-chamber view.

Leaflet motion abnormalities can be described as classically defined by echocardiography (prolapse, flail) (Figure 1). However, a flail leaflet in CMR can be masked by the regurgitant flow. Maximum mitral leaflet thickness is measured in diastole. Standard long-axis cine images are also informative for visualizing and sizing the mitral annulus (14). With CMR reference ranges, mitral annular dilatation, one of the mechanisms in secondary MR, can be identified. The mitral annular diameter is measured from the anterior commissure to the posterior commissure in the diastole (Figure 1). In primary MR (mitral valve prolapse especially), CMR can detect mitral annulus disjunction, a frequent component of myxomatous mitral valve disease (15) using standard long-axis cine images.

The LVOT-1 view can be used for determining the MV leaflet length, MV tenting, and tenting height, major determinants of ischemic MR. However, there are no data comparing CMR measurement of the mitral annulus or MV length to surgical findings, and echocardiography remains the gold standard in the perioperative assessment of primary MR.

##### Technical Considerations

With a slice thickness between 5 and 7 mm, imaging the length and thickness of the mitral chordae is less accurate than echocardiography. In addition, since CMR images are reconstructed over several heartbeats, thin and very mobile structures such as vegetation, cordal rupture, or fibroelastoma are often poorly visualized when their motion is variable in the cardiac cycle. Moreover, due to lower spatial resolution than echocardiography (especially transesophageal echocardiography), annular or leaflet calcifications seen with a slightly darker-than-myocardium signal in bSSFP sequences cannot be properly identified by CMR (16). Thus, echocardiography (transthoracic and/or transesophageal) is still the gold standard for the assessment of MV morphology and MR mechanisms.

#### 2D Cine Imaging for Regurgitant Jet Visualization

On cine images, regurgitant jets can be visualized as a signal loss (dark/low signal) created by the turbulence of flow in the left atrium. The jet can be difficult to be clearly identified on fixed 2D images, especially if the direction of the jet is not linear. Moreover, the size and length of the jet can be reduced or increased by just changing parameters or sequences. Rapid spoiled gradient-recalled echo sequences with longer repetition and echo times or gradient echo (GRE) or hybrid GRE echo-planar sequences have been proposed for higher sensitivity (13, 17). The identification of such a flow void provides gross information about the location and direction of the jet, which can help in determining the etiology of the MR (for example, a central or eccentric jet, suggesting secondary or MV prolapse, respectively) (Figure 1). Compared to gradient-echo imaging, SSFP imaging results in significantly reduced signal voids in regurgitant jets in general and can easily underestimate the degree of regurgitation. These sequences are much less sensitive for identifying regurgitation than echocardiography and do not represent a reliable method for evaluating MR severity.

### Cardiovascular Magnetic Resonance for Quantifying Mitral Regurgitation

There are several CMR methods for evaluating MR severity; all are independent of the characteristics of the mitral regurgitant jet and do not require calculation using a complex equation. These methods are less accurate in arrhythmic patients or poor breath-holders. A free-breathing phase-contrast sequence with an increased number of averages (≥3) can be applied in these cases. The methods available in clinical practice are summarized in Table 1.

**TABLE 1:** Various CMR methods used in clinical practice to quantify MR, with their advantages and limitations.

	Indirect		Direct
CMR method	1: Recommended	2	Not recommended
CMR sequences required	SSFP short-axis images for LVSV	Volumetric SSFP short-axis images for LVSV and RVSV	Long-axis cine SSFP images and phase-contrast velocity mapping placed on the atrial side of the MV, perpendicular to the direction of the jet
	Phase-contrast velocity mapping at the sinotubular junction for AFF In aortic stenosis: Phase-contrast velocity mapping of the main pulmonary artery for PFF		
Formula	MRvol (ml) = LVSV—AFF RF (%) = MRvol/LVSV	MRvol = LVSV—RVSV	Direct quantification of MRvol
	In aortic stenosis: MRvol = LVSV—PFF		
	In aortic regurgitation: MRvol = LVSV- (AFF + ABF)		
Advantages	Reproducible Independent of jet morphology Valid for patients with multiple regurgitation	Fast and simple	Valid for patients with multiple regurgitations or intracardiac shunts
Limitations	Careful basal slice selection at the LV base for systolic volume in mitral prolapse Careful perpendicular slice selection Phase-offset errors Ensure correct maximal velocity encoding	Inaccurate for patients with multiple regurgitations and intracardiac shunts Careful basal slice selection of LV and RV	High velocity jets can cause spin dephasing and displacement artifacts Difficult to select the plane position for patients with eccentric jets with mobile valves Inaccurate for patients with high heart rate variability

*CMR, cardiac magnetic resonance; SSFP, steady-state free precession; LVSV, left ventricular stroke volume; RVSV, right ventricular stroke volume; AFF, aortic forward flow; ABF, aortic backward flow; RF, regurgitant fraction; PFF, pulmonary forward flow; MRvol, mitral regurgitant volume.*

#### Indirect Method n°1: 2D Cine Imaging and 2D Cine Phase-Contrast Velocity Mapping

The most widely used CMR method to quantitatively assess the severity of MR is an indirect method combining 2D cine imaging and phase-contrast velocity mapping to quantify the regurgitant volume and fraction.

Mitral regurgitant volume (MRvol) is expressed as the difference between the left ventricular stroke volume (LVSV) and aortic forward flow (AFF), and the regurgitant fraction (RF) as the MRvol divided by the LVSV, expressed as a percentage.

**FIGURE 2:**
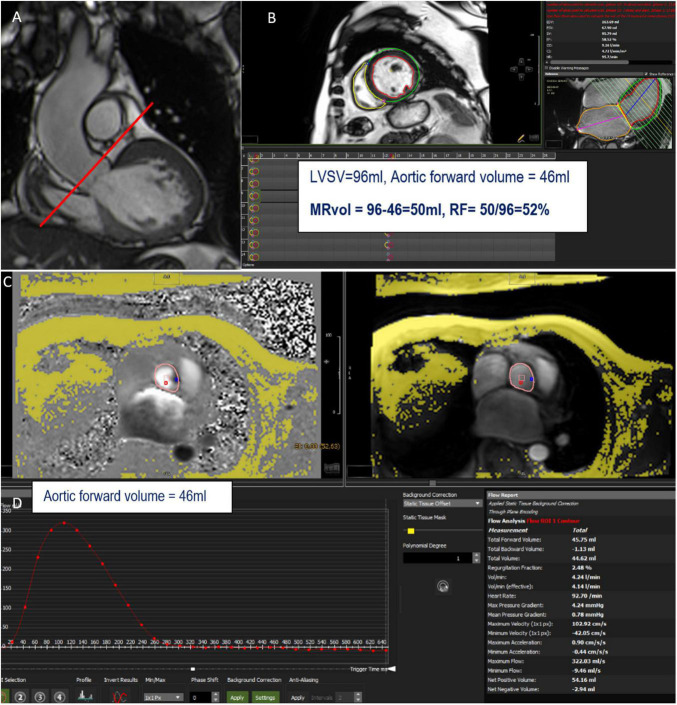
Example of mitral regurgitation (MR) assessment using indirect method n°1. LVOT bSSFP images show the perpendicular line above the aortic valve (red line) indicating the slice position for phase-contrast velocity mapping **(A)**. Assessment of left ventricular stroke volume (LVSV) from short-axis cine SSFP images **(B)**. Phase and magnitude images with delineation of the aorta **(C)** generating flow curves **(D)**. This patient has a severe MR with a mitral regurgitant volume (MRvol) of 50 ml and a regurgitant fraction (RF) of 52%.

**FIGURE 3:**
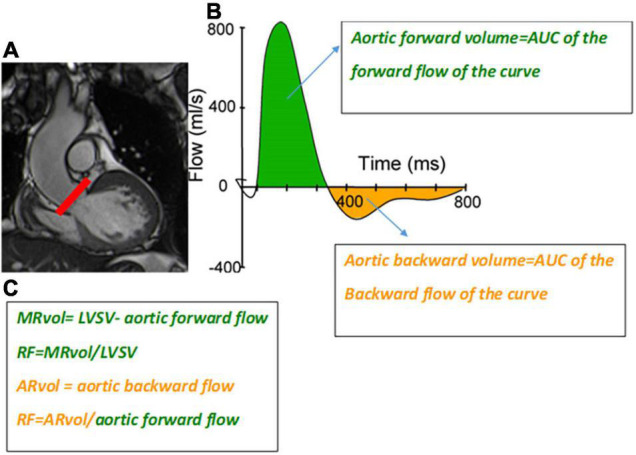
**(A)** (left) Slice position for the phase-contrast velocity mapping sequence. **(B)** Flow curve (mL/s) through the aorta at the level of the line on panel **(A)**. Aortic forward volume is calculated as the area under the curve (AUC) of the forward flow on the flow curve (green area); aortic backward volume is calculated as the AUC of the backward flow on the flow curve (orange area). **(C)** Formula to obtain mitral and aortic regurgitant volume (MRvol and ARvol) and regurgitant fraction (RF) are shown at the left bottom.

The LVSV can be obtained from multiple LV short-axis cine bSSFP images, and the AFF from phase-contrast velocity flow in a trough-plane acquired at the level of the sinotubular junction or the aortic valve but not at the mid-ascending aorta due to the risk of overestimation (18). This sequence generates two types of images: a magnitude image showing the anatomy of the aortic valve and a phase map encoding the velocities within each voxel. A dedicated post-processing analyzes the blood flow through the aortic plane generating a flow curve, which allows the calculation of the aortic forward flow (AFF) (Figures 2, 3). An average of two to three flow measurement acquisitions is optimal.

##### Advantages

This method is highly reproducible (19, 20), independent of the number, shape, or morphology of regurgitant jets (particularly useful for patients with eccentric or multiple jets), and is not affected by tricuspid or pulmonary regurgitation. In cases with concomitant aortic regurgitation, aortic diastolic flow (aortic backward flow: ABF), obtained directly from the diastolic flow of the aortic phase-contrast image, must be added to the AFF in the equation (Table 1) (17).

In cases with aortic stenosis, the through-plane flow imaging slice is placed beyond the turbulent jet. In exceptional cases, the pulmonary forward flow (PFF), instead of the AFF due to non-laminar and high aortic velocity in the ascending aorta, can be used.

##### Technical Considerations

Technical considerations are detailed in Table 1. Although CMR is the gold standard for volumetric assessment, variability may arise due to basal slice selection for LVSV measurement which can be challenging in patients with MR due to prolapse with Barlow disease, with increased annular excursion due to mitral annular disjunction (21). Accordingly, we suggest measuring the LV end systolic volume at the base of the LV rather than at the leaflets in these patients, resulting in a better assessment of LV function and MR severity, with higher LV ejection fraction (EF), MRvol, and RF.

The accuracy of the AFF may be compromised by potential errors:

- Mismatch of encoding velocity: the velocity encoding value (Venc) should be set within 25% of the true maximal velocity in the aorta to avoid aliasing.
- Misalignment of imaging plane: to avoid inaccurate aortic peak velocity, the imaging plane should be orthogonal to the direction of flow.
- Phase offset errors, due to local magnetic field inhomogeneities, can be corrected by using a background correction.
- During analysis, noise pixels should not be included in the contouring of the aorta.

Validation of AFF by CMR has been established by comparison to left ventricular stroke volume with echocardiography with good correlation (Table 2) (22, 23). Inter- and intra-observer variability of AFF, indirectly assessed in patients with AR by measuring RF (= ARvol/AFF, Figure 3), is very good with an excellent correlation coefficient (0.956 and 0.998 respectively) and small standard errors of the estimate (1.19% and 0.34% respectively) (24).

**TABLE 2:** Validation of forward flow in the aorta by comparison to echocardiography.

First author, year (Ref #)	Number of patients	Reference method	Correlation coefficient
Bogren et al. (22)	24 normal patients	Left stroke volume in echocardiograhpy	0.93
Van Rossum et al. (23)	17 healthy volunteers	Left stroke volume in echocardiograhpy	0.76

#### Indirect Method n°2: Volumetric Method: 2D Cine Imaging

An alternative and simple approach using only bSSFP sequences in the short axis is the “volumetric method,” expressed as the difference between LVSV and right ventricular stroke volume (RVSV), which represents the quantity of MR in the absence of other types of valvular regurgitation or intracardiac shunts.

##### Advantages

Using a single method is less prone to measurement error than using two different techniques.

##### Technical Considerations

Due to the shape of the right ventricle (RV) and extensive trabeculations, RV contouring is more prone to errors in RVSV measurements.

#### Direct Method

Direct quantification of MR flow is theoretically feasible with phase-contrast velocity mapping by directly measuring the regurgitant flow across the MV.

However, given the significant dynamic motion of the MV during systole, high velocity, a non-circular orifice, and jet angulation, positioning a fixed slice across the MV (without annular valvular tracking) can be challenging and inappropriate, especially for patients with eccentric MR, multiple jets with different directions, and high heart-rate variability. Therefore, we do not recommend this approach for clinical practice.

#### 4D Flow

4D flow velocity-encoded CMR imaging is an emerging technique that involves phase-contrast acquisition with flow encoding in all three spatial directions and to the dimension of time (3D + time = 4D). It allows the visualization of flow in multiple orientations to follow the spatial motion of the heart valves and the variable direction of the jets over time. The use of such a retrospective tracking method in a single volume acquisition covering the entire heart may enable direct MR jet volume quantification, overcoming certain limitations of 2D sequences (25). Recent reports have demonstrated its feasibility in mild to severe MR (26). However, its accuracy has usually been poor in comparison to 2D flow, especially in primary MR (25). This promising technique, which is constantly improving, is not yet commercially available for all scanners. Its principal disadvantage is the long scanning time (up to 10 min). Furthermore, it requires a post-processing software. Another limitation for direct measurement of MR severity is also the high velocity of the MR jet (> 5 m/s), which may result in aliasing and which can be more difficult to correct in 4D flow imaging and may require multiple velocity encodings.

#### Late Gadolinium Enhancement Imaging and T1 Mapping for Additional Information

Late gadolinium enhancement (LGE) imaging, acquired after gadolinium administration, has the unique ability to provide tissue characterization of the myocardium by assessing and quantifying regions of fibrosis and scars across the left atrium (27), the LV (28), and the papillary muscle (29).

Late gadolinium enhancement sequences [pulse sequences according to published guidelines (12)] should be performed at least 10 min after gadolinium injection in the short axis and the three long-axis planes (13). High-resolution dark blood LGE CMR is required to identify small papillary muscle enhancement (30).

In ischemic MR, the quantification of MR and myocardial infarct size with CMR can help for risk stratification in patients with advanced ischemic cardiomyopathy (31). In primary MR, especially MV prolapse, a high prevalence of focal replacement fibrosis, particularly in the segments adjacent to the posterior papillary muscle, can be observed (29). However, the LGE technique is not able to characterize diffuse fibrosis due to the absence of a normal myocardium as the reference. A recent parametric mapping technique (T1 mapping) allows for better characterization of global myocardial tissue composition by direct measurement of T1 relaxation times before and after contrast administration (32) and by calculation of the extracellular volume (ECV) (33).

In MR, little is known about diffuse interstitial fibrosis. One recent study suggested that in primary MR, ECV increases with MR severity, regardless of MV prolapse or the presence of replacement fibrosis (34). Diffuse interstitial fibrosis in primary MR may be more closely related to chronic LV volume overload than the etiology of MR.

### Assessment of MRVol by Echocardiography and Cardiovascular Magnetic Resonance

Quantification of MR by echocardiography is based on 2D echocardiography, color flow, and Doppler parameters. Current echocardiographic guidelines consider severe MR as MRvol ≥ 60 ml (or effective regurgitant orifice ≥ 40 mm^2^) by the PISA method and RF ≥ 50% (35). Strikingly, the echocardiographic cut-off of MRvol ≥ 60 ml and or RF ≥ 50% were also considered for severe MR in the vast majority of CMR studies and the current guidelines (35, 36) (Table 3).

**TABLE 3:** Cutoff of CMR parameters to define severe MR and AR according to current American (36) and European recommendations (35).

	Severe MR	Severe AR
ASE recommendations (36)	RF ≥ 50% MRvol ≥ 60 ml	RF ≥ 50% ARvol ≥ 60 ml
ESC recommendations (35)	RF ≥ 50%	RF ≥ 50%

*CMR, cardiac magnetic resonance; MR, mitral regurgitation; AR, aortic regurgitation; ASE, American Society of Echocardiography; ESC, European Society of Cardiology; RF, regurgitant fraction; MRvol, mitral regurgitant volume; ARvol, aortic regurgitant volume.*

#### Comparison Between Echocardiographic MRvol and Cardiovascular Magnetic Resonance MRvol

Most studies have shown only limited agreement in the assessment of MR between CMR and echocardiography (20, 37–39). MRvol values truly differ among volumetric methods and the PISA method. There is a consistent tendency to obtain higher MRvol values by PISA relative to CMR (40–42), resulting in higher MR severity assessment with a risk of undergoing surgery without severe MR by CMR (20). The discrepancy between these two modalities is more pronounced in patients with late systolic or multiple jets than central and holosystolic jet (38). Therefore, using the same thresholds to define MR severity and the need for surgery may be inappropriate. The present available literature indicates that PISA MRvol thresholds and MRvol values by CMR are different and not interchangeable. Interestingly, the MRvol was similar using the volumetric method by 3D echocardiography and CMR (43). Recent reports have suggested that 3D-transthoracic echocardiography is less dependent on geometric assumptions and allows better visualization of the vena contracta regurgitant orifice. However, characterization of the Doppler jet is still challenging, with additional limitations of lower spatial and temporal resolution (36).

#### Cardiovascular Magnetic Resonance to Identify Significant MR and for the Timing of Intervention

There is a paucity of data on specific CMR thresholds to define MR severity due to the lack of large trials with a validation cohort. Gelfand et al. (44) proposed that RF CMR cutoff value of 42% (indirect method) for severe MR correlates well with Doppler echocardiography. Interestingly, one study used the multiparametric approach by Doppler echocardiography as the reference standard and compared it to CMR RF. MR severity graded by two experts, showed excellent agreement. The authors observed that significant MR (moderate to severe or severe) could be very accurately identified by CMR using a RF cutoff value of 35% (45).

Myerson et al. (46), on initially asymptomatic patients with moderate or severe MR followed up for up to 8 years, showed that MRvol and RF by CMR were the most discriminatory parameters to determine the need for surgery (symptoms or other indications for surgery), with cut-off thresholds of 55 ml for MRvol and 40% for RF, and an increasing risk with increasing values of the parameters. The RF cutoff for severe MR in this study was very similar to a recent study by Polte et al. (47) (RF > 41%) and lower than echocardiographic criteria (RF ≥ 50%). As the MRvol is proportional to LV size in primary MR, for two patients with similar CMR MRvols, the patient with a smaller LV will have more severe MR; calculation of the RF may overcome this issue as it normalizes the MRvol to the size of the LV.

Interestingly, integrating ECV may also provide an additional benefit for the selection of asymptomatic patients for mitral correction. Recently, Kikungvan et al. (48), in a prospective observational registry of patients with at least moderate primary MR, showed that RF and elevated ECV were independently associated with events, with a cutoff of 40% for RF and 30% for ECV for mitral surgery.

Another approach to evaluate the accuracy of an imaging modality to quantify MR is to assess LV remodeling after surgery. Some authors have found a good correlation between MRvol by CMR and a decrease in LV volume post-MR correction (20, 40).

##### Main Message for the Clinician

Based on the most recent guidelines (4), echocardiography remains the first-choice tool to grade MR based on qualitative, semiqualitative, quantitative, and structural criteria. Current class I surgical recommendations for severe MR are based on symptoms or, for asymptomatic patients, on LV dilatation or dysfunction (LVESD ≥ 40 mm and/or LVEF ≤ 60%). CMR is a “second tool,” indicated when various echocardiographic parameters are inconsistent (Table 4). Based on the relevant publications discussed in this review illustrating the accuracy of CMR to assess LV volume, MR severity, and predict LV reverse remodeling after correction (Tables 5, 6), this imaging modality should be considered not only for patients with severe MR by echocardiography to confirm the severity and help guide surgical decision-making but also for patients for whom the severity of MR is unclear by echocardiography. In light of studies discussed above, MR is very likely to be severe if the mitral RF is ≥ 40% of CMR, even if large trials with a validation cohort to define the best CMR cutoff are needed.

**TABLE 4:** Indications, preferred methods and evidence level of CMR to determine severity of mitral, aortic, tricuspid and pulmonary regurgitation according to current American and European guidelines (3, 4, 35, 36).

	Severe MR	Severe AR	Severe TR	Severe PR
When CMR is indicated	Discrepancy between MR severity on echo and clinical findings Inconclusive echo on MR severity or indeterminate MR (3, 4, 35, 36)	Discrepancy between AR severity on echo and clinical findings Inconclusive echo on AR severity or indeterminate AR (3, 4, 35, 36) Patients with bicuspid aortic valve with unsatisfactory assessment of aorta morphology (36)	Discrepancy between TR severity on echo and clinical findings Inconclusive echo on TR severity or indeterminate TR (3, 4, 35, 36) RV assessment (35)	Discrepancy between PR severity on echo and clinical findings Inconclusive echo on PR severity or indeterminate PR (3, 35, 36) RV assessment (35)
Preferred CMR methods	Indirect methods (35, 36)	Direct method (35, 36)	Indirect methods (35, 36)	Direct method (35, 36)
Class of recommendation/Level of evidence	I/B-NR (3)	I/B-NR (3)	I/B-NR (3)	–

*CMR, cardiac magnetic resonance; MR, mitral regurgitation; AR, aortic regurgitation; TR, tricuspid regurgitation; PR, pulmonary regurgitation; NR, not randomized.*

**TABLE 5:** Relevant studies on validation of CMR parameters in MR and AR.

First author, year (Ref #)	Number of patients	Reference method	Optimal CMR parameters cutoff for concordance with echo or for severe regurgitation (sensibility/specificity)
**Mitral regurgitation**			
Polte et al. (47)	40	Indication for surgery based on echo recommendation	RF > 41% (96%/80%) MRvol > 64 ml (96%/87%)
Le Goffic et al. (45)	34	TTE based on integrative approach	RF > 35% (86%/100%)
Gelfand et al. (44)	83	TTE (qualitative)	RF > 42% (NA)
**Aortic regurgitation**			
Polte et al. (47)	38	Indication for surgery based on echo recommendation	RF > 30% (87%/67%) ARvol > 40 ml (87%/73%)
Gabriel et al. (62)	107	TTE based on ASE guidelines	RF ≥ 30% (NA)

*CMR, cardiac magnetic resonance; MR, mitral regurgitation; AR, aortic regurgitation; RF, regurgitant fraction; MRvol, mitral regurgitant volume; ARvol, aortic regurgitant volume; TTE, transthoracic Echocardiography; ASE, American Society of Echocardiography; NA, not available.*

**TABLE 6:** Relevant studies assessing prediction of outcome of severe MR and AR assessed by CMR.

First author, year (Ref #)	Number of patients	Follow up	Primary outcome	Optimal CMR parameters cutoff for primary endpoint (sensibility/specificity)
**Mitral regurgitation**				
Cavalcante et al. (31)	578 (ischemic MR)	Median at 4.9 years	All-cause mortality or heart transplantation at 1 year	RF ≥ 35% (NA/84%)
Myerson et al. (46)	109 (organic MR)	Mean 2.5 ± 1.9 years	Development of indications for surgery	RF > 40% (76%/74%) MR vol > 55 ml (72%/87%)
**Aortic regurgitation**				
Faber et al. (69)	66	Median 5.1 years	Prediction of valve surgery	RF > 32% (NA)
Harris et al. (64)	29	Mean 4.4 ± 1.5 years	Need for valve surgery and heart failure	RF ≥ 37% (100%/75%) ARvol ≥ 50 ml (100%/NA)
Myerson et al. (68)	113	Mean 2.6 ± 2.1 years	Development of indications for surgery	RF > 33% (85%/92%)

*CMR, cardiac magnetic resonance; MR, mitral regurgitation; AR, aortic regurgitation; RF, regurgitant fraction; MRvol, mitral regurgitant volume; ARvol, aortic regurgitant volume; NA, not available.*

## Aortic Regurgitation

Aortic regurgitation (AR) is the second most frequently occurring type of regurgitation after MR (49). As for MR, echocardiography is the first-line modality for AR assessment, and CMR is recommended as a complementary technique when image quality is suboptimal or in situations of discordance results (3, 4). However, dynamic or multiple jets, bicuspid valve or aortic valve calcifications, a non-hemispheric shape, or multivalvular disease are among the echocardiographic limitations that can affect the accuracy of AR assessment. Below, we describe how CMR can help in defining the etiology, assessing the consequences of AR on LV remodeling, grading AR severity, and defining the timing for AR correction.

### Cardiovascular Magnetic Resonance for Assessing the Cause of Aortic Regurgitation

#### 2D Cine Imaging for LV Volume and Aortic Valve and Root Morphology

**FIGURE 4:**
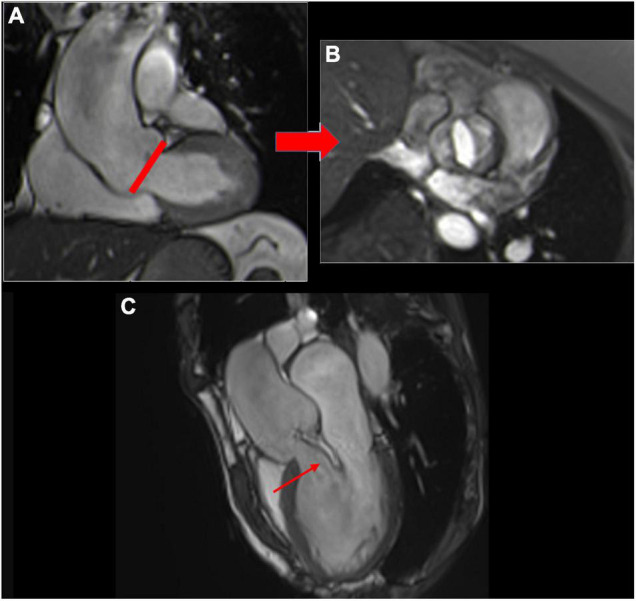
Coronal oblique bSSFP sequence through the LVOT **(A)**; red line indicates image plane for direct visualization of the aortic cusps **(B)** showing a true bicuspid aortic valve (without raphe). Eccentric aortic jet (flow void in the left ventricle, red arrow) visualized in the coronal LVOT view **(C)**.

With a high contrast between the valve leaflets and blood pool, together with a high signal-to-noise ratio, SSFP imaging can be used to visualize aortic valve anatomy in any plane, irrespective of cardiac anatomy (50). A perpendicular plane through the coronal oblique LVOT view allows the visualization of each cusp (left, right, and non-coronary). Contiguous cine images 3 mm above and below this slice are required (51). During diastole, three normal leaflets form a three-pointed star “Mercedes-Benz emblem,” but a bicuspid aortic valve can be identified (Figure 4).

Assessing the aortic diameter is crucial for determining AR etiology and follow-up. The diameter of the ascending aorta can be measured using non-contrast-enhanced MR angiography (NCE-MRA) with a respiratory navigator or breath-hold contrast-enhanced MRA (CE-MRA). Measurements are performed at end-diastole using the internal diameter of the aorta at the level of the sinus, the sinotubular junction, and the level of the pulmonary trunk on the ascending aorta (52) or at the level of maximum dilatation. Ideally, measurement should be performed in 3D using a double oblique angulation perpendicular to the vessel axis. Measurement in standard 2D axial view should be avoided.

#### 2D Cine Imaging for Regurgitant Jet Visualization

Aortic regurgitant jets can be visualized on cine images using long-axis LVOT views (3-chamber and coronal oblique LVOT) as a dark jet due to a signal void from turbulence back flowing into the LVOT during diastole (Figure 4). The location and direction of the jet can provide additional information about the AR etiology. For example, a central jet can be more highly related to aortic dilatation and an eccentric jet more to a cusp abnormality (prolapse) (Figure 4). However, the appearance of the signal void is dependent on the hemodynamics, echo time, and flip angle (53), and does not directly reflect the aortic regurgitant volume (ARvol); therefore, a more reliable assessment is required.

### Cardiovascular Magnetic Resonance for Quantifying Aortic Regurgitation

Various CMR methods used in clinical practice to assess AR severity are summarized in Table 7.

**TABLE 7:** Various CMR methods used in clinical practice to quantify AR, with their advantages and limitations.

CMR method	Direct recommended	Indirect	
		1	2
		Phase contrast mapping	Volumetric
CMR sequences required	Phase-contrast velocity mapping above the aortic valve Phase-contrast velocity mapping in the descending aorta for HFR	Phase-contrast velocity mapping of the proximal aorta and the main pulmonary artery for AFF and PFF	SSFP short-axis images for LVSV and RVSV
Formula	Direct quantification ARvol (ml) = area under the diastolic flow curve RF (%) = ARvol/AFF	ARvol = AFF—PFF	ARvol = LVSV—RVSV
Advantages	Reproducible, particularly for laminar jets Valid for multiple regurgitation Fast post-processing	Rapid	Rapid and simple
Limitations	Less accurate for no laminar jets and in cases of associated aortic stenosis Careful placement 5 mm above the aortic valve perpendicular to the jet is necessary Phase-offset errors Ensure correct maximal velocity encoding	Inaccurate for multiple regurgitations Careful perpendicular slice selection Phase-offset errors Ensure correct maximal velocity encoding	Inaccurate for multiple regurgitations Careful basal slice selection for LV and RV

*CMR, cardiac magnetic resonance; SSFP, steady state free precession; LVSV, left ventricular stroke volume; RVSV, right ventricular stroke volume; AFF, aortic forward flow; RF, regurgitant fraction; PFF, pulmonary forward flow; ARvol, aortic regurgitant volume; HFR, holodiastolic flow reversal.*

#### Direct Method: 2D Cine Phase-Contrast Velocity Mapping

**FIGURE 5:**
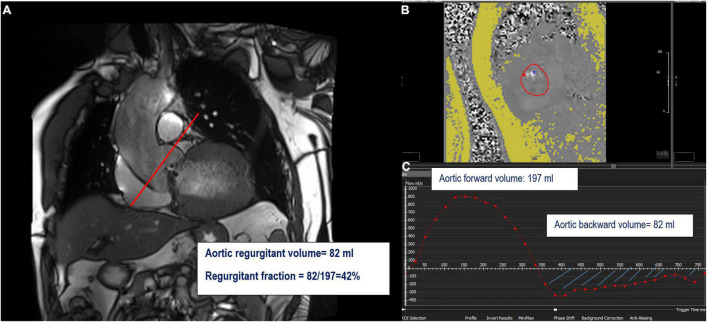
Example of the direct quantification of aortic regurgitation by phase-contrast imaging. Perpendicular line (red) above the aortic valve on LVOT view indicating the slice position for phase-contrast velocity mapping **(A)** generating phase images **(B)** and flow curves images **(C)**. The aortic regurgitant volume (ARvol) is represented by the area under the diastolic flow curve (blue hatch lines).

Cardiovascular magnetic resonance has the ability to measure flow velocity and direction over time. Unlike MR (with multiple, turbulent, irregularly shaped jets), direct flow quantification of AR is feasible. The most commonly used CMR method to quantitatively assess the severity of AR is a direct measurement using through-plane velocity mapping performed just above the aortic valve (2D phase-contrast imaging: 2D-PC) in a plane perpendicular to the direction of blood flow (21). A dedicated post-processing analyzes the blood flow through the aortic plane generating a flow curve, which allows calculation of the AFF, aortic regurgitant volume (ARvol = area under the backward flow curve during the diastolic phase of the cardiac cycle), and RF (ARvol/AFF) (54) (Figures 3, 5).

##### Advantages

This method is highly reproducible and accurate, especially for laminar regurgitant flow, with an imaging plane perpendicular to the aorta (55). It requires only a single breath-hold, is valid in cases of coexisting valvular lesions, and post-processing is rapid.

##### Technical Considerations

An incorrect velocity encoding setting (below the peak velocity in the vessel) results in aliasing and an underestimated peak velocity (56). We recommend starting with an encoding velocity slightly below the peak velocity of AR found in echocardiography and repeating the flow measurement with a higher encoding velocity until it corrects the aliased data set. In the presence of associated, aortic stenosis, the peak systolic velocity can be underestimated due to lower temporal resolution relative to echocardiography, leading to an underestimation of the AFF.

##### Additional Parameter

According to recent CMR guidelines (12), velocity-encoded imaging in a through-plane perpendicular to the descending aorta is recommended to explore for diastolic flow reversal, similar to echocardiography (35). In, Bolen et al. (57) found that HFR (≥ 10 ml/s) is highly sensitive (100%) and specific (93%) in predicting severe AR. More recently, a study by Kammerlander et al. showed an association between HFR and increased cardiovascular events (58).

#### Indirect Method n°1: 2D Cine Phase-Contrast Velocity Mapping

ARvol can be measured indirectly as the difference between the AFF and PFF in the absence of other regurgitation or intracardiac shunts.

##### Advantages

This method requires only 2 single breath-holds and allows for rapid post-processing. If there are concerns about the direct method, this method can serve for internal validation.

##### Technical Considerations

This method is not valid in situations of pulmonary regurgitation or intracardiac shunts.

#### Indirect Method n°2: Volumetric Method: 2D Cine Imaging

Similar to MR, the volumetric method can be used to quantify ARvol using only bSSFP sequences in the short axis. ARvol is expressed as the difference between LVSV and RVSV in the absence of other types of valvular regurgitation (54).

##### Advantages

This method is simple and does not require specific acquisitions because bSSFP cine sequences are performed during every CMR examination.

Technical considerations are described in the MR chapter.

#### 4D Flow, Late Gadolinium Enhancement Imaging, and T1 Mapping

4D flow with its ability for measuring eccentric, non-laminar flow in any orientation in space could be in the near future appropriate for AR quantification (59).

T1 mapping, with its potential to assess cellular and extracellular compartments, could be of interest in AR as a prognostic marker for clinical outcomes (60).

### Comparison of Cardiovascular Magnetic Resonance and Echocardiography on ARvol and Aortic Regurgitation Severity

#### Modest Correlation With Echocardiography, Particularly for Eccentric Jets

Unlike MR, there is a paucity of large and prospective studies comparing these two modalities. In all studies, the direct CMR method was used and thresholds to define severe AR were the same as those used for echocardiography: Rvol ≥ 60 ml and RF ≥ 50% according to recommendations (35, 36, 61) (Table 3). In most studies, the correlation between these two modalities was modest (62–65), particularly in eccentric jets (66).

2D PISA is limited to the alignment of the flow and by the need for computation and geometric assumptions. 3D echocardiography, not restricted by any imaging plane, could overcome these limitations and provide a better correlation with CMR, especially in eccentric (67).

#### Cardiovascular Magnetic Resonance for Grading Severe Aortic Regurgitation and for the Timing of Surgery

As previously noted, ARvol and/or RF measured by CMR are categorized based on established echocardiographic guidelines (35), defined as:

- Mild to moderate AR: 30 mL ≤ ARvol < 45 ml,
- Moderate to severe AR: 45 mL ≤ ARvol < 60 ml,
- Severe AR: ARvol ≥ 60 ml; RF ≥ 50%

As for MR, there is a paucity of data on specific CMR thresholds for ARvol and RF, with a lack of large trials with a validation cohort. ARvol > 40 ml and RF ≥ 30% have been proposed to define severe AR with CMR that correlates best with echocardiography (47, 62) or for identifying patients who developed symptoms or needed aortic correction surgery (68). These CMR thresholds are much lower than established echocardiographic guideline criteria (Rvol ≥ 60 ml and RF ≥ 50%) (35, 36) and could explain the frequent mismatch between moderate to severe AR by 2D echocardiography and mild to moderate AR by CMR (65).

In the absence of a reference method, it is difficult to determine which imaging modality can better predict the need for aortic correction, particularly for asymptomatic patients. However, several studies have reported a better prediction with CMR (64, 65, 69).

#### Main Message for the Clinician

Echocardiography is the first-choice tool to grade AR based on qualitative, semiqualitative, and quantitative criteria (4). Current class I surgical recommendations for severe AR are based on symptoms or for asymptomatic patients with LV dilatation or dysfunction (LVESD ≥ 50 mm or > 25 ml/m^2^ or LVEF ≤ 50%). CMR is a “second tool,” indicated when various echocardiographic parameters are inconsistent (Table 4). Although there is a lack of large, prospective, comparative studies, the publications discussed above show that CMR could improve the diagnosis and surgical timing of patients with AR relative to echocardiography (Tables 5, 6). CMR should be considered for patients with severe AR by echocardiography to confirm the severity and guide surgical decision-making. From a practical point of view and in light of studies discussed above, AR is very likely to be significant if aortic RF ≥ 30%, even if large trials to define specific CMR thresholds are needed.

## Tricuspid Regurgitation

Unlike MR and AR, less data are available about the prevalence of moderate to severe tricuspid regurgitation (TR), which is probably underestimated. In a recent study performed in the United States (Olmsted County, Minnesota), the age and sex-related prevalence of TR (diagnosed by Doppler echocardiography) was 0.55%, higher among women, and increased with age (4.4% among women aged ≥ 75 years and 3.1% among men) (70). Moderate to severe TR is associated with adverse outcomes, independent of LVEF or pulmonary artery pressure (71), probably due to the development of right-heart failure.

Causes of TR can classically be divided into two types:

- Primary TR due to a primary lesion of the tricuspid valve caused by congenital or acquired disease.
- Secondary (or functional) TR, with a structurally normal valve, more commonly associated with left-sided heart disease, pulmonary hypertension, RV dysfunction, dilatation, or without a detectable cause and thus called isolated (or idiopathic) TR, but often associated with atrial fibrillation.

Whether isolated TR or not, understanding TR, its consequences, and accurately assessing its severity is crucial, particularly since the emergence of transcatheter interventions, which have moved the tricuspid valve from the shadows into light. Echocardiography is the preferred and recommended initial imaging technique to assess TR based on an integrative approach, including qualitative and quantitative criteria. The use of conventional quantitative parameters (vena contracta and PISA) is based on geometrical assumptions (circular jet and flat orifice) extrapolated from the MV (72). However, due to the complex non-planar geometry of the tricuspid annulus, these assumptions may not be valid for the tricuspid valve (73). Although there are few studies that have assessed TR by CMR, this modality has the unique advantage of non-invasively measuring the flow through vessels and to be the reference standard modality for RV volume and function assessment, important parameters to take into account in TR. In particular, CMR allows a more precise evaluation of RV volumes and EF than the echo.

### Cardiovascular Magnetic Resonance for Assessing Tricuspid Regurgitation

#### 2D Cine Imaging for RV Volume and Tricuspid Valve Morphology

Due to the complex interplay between TR and the RV (TR can cause RV dilatation and vice versa), assessing TR requires the accurate assessment of RV structure and function. CMR is the method of reference to assess RV volume, function, and mass, without geometric assumptions (74).

RV structure can be studied using

**FIGURE 6:**
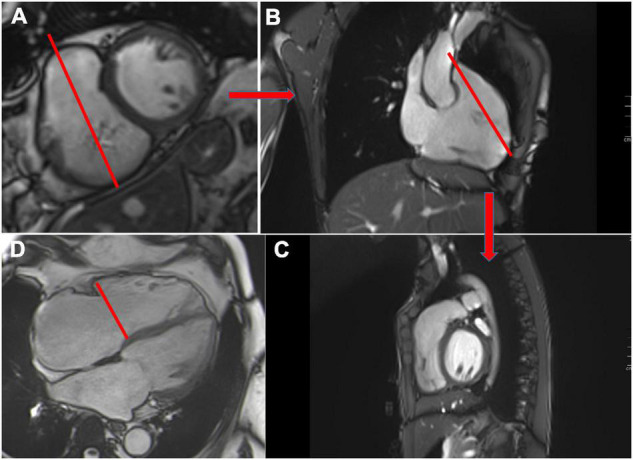
Cardiovascular magnetic resonance planes used for evaluation of the right ventricle. Short axis view **(A)**, 3-chamber view **(B)** and RVOT **(C)**. Example of tricuspid annular measurement in the 4-chamber view (red line) **(D)**.

- a stack of contiguous bSSFP short-axis images, the same as those used for LV, with careful placement of the basal slice on the myocardial side of the RV (75). In cases of complex congenital disease, dedicated axial orientation on the RV (slice thickness between 5 and 6 mm without interval gaps) is recommended (36).
- Long-axis cine images, including three specialized views for the RV: RV out-flow tract (RVOT) and RV vertical long axis (3-chamber) (Figure 6).

The RV regional wall-motion abnormalities can be determined visually. Recently, feature tracking, similar to speckle tracking in echocardiography, has emerged to more accurately detect changes in wall motion and earlier ventricular dysfunction, which may be an independent predictor of mortality in severe TR (76).

Tricuspid anatomy can be assessed by short- and long-axis views, as already described, allowing simultaneous visualization of the three leaflets (septal, anterior, and posterior) in the short-axis view. Leaflets can be described as prolapsed, restricted, thickened, or with tenting (77). Tenting height and area may be measured and considered abnormal if they are > 7 mm (3 mm/m^2^) and 1.1 cm^2^ (> 0.5 cm^2^/m^2^), respectively (14). However, tricuspid leaflets are thin, as well as the chordae and papillary muscles, and can be difficult to visualize. Therefore, echocardiography remains the cornerstone for TR anatomy assessment.

The tricuspid annular diameter, a relevant parameter included in the guidelines for tricuspid intervention, can be measured in the 4-chamber view in early diastole, with the upper limits of the normal value recently reported to be 43 mm (22 mm/m^2^) (78) (Figure 6). It is important to note that for patients with pacemakers or defibrillator leads, generated metallic artifacts decrease the accuracy of CMR for the assessment of RV volume structure and tricuspid anatomy, as well as for patients with arrhythmias.

#### 2D Cine Imaging for Regurgitant Jet Visualization

The qualitative assessment of TR can be performed using bSSPF imaging (2 specialized long-axis and 4-chamber views). TR is visualized as a dark jet created by flow turbulence generating spin dephasing back flowing into the right atrium. As for MR and AR, this is a gross assessment and is not reliable on its own. In the presence of a turbulent jet, regurgitant flow appears as a low signal that can mask the visualization of the leaflets.

### Cardiovascular Magnetic Resonance for Quantifying Tricuspid Regurgitation

Quantitative assessment of TR by CMR can be performed using two indirect and one direct method.

#### Indirect Method n°1: 2D Cine Imaging and 2D Cine Phase-Contrast Velocity Mapping

**FIGURE 7:**
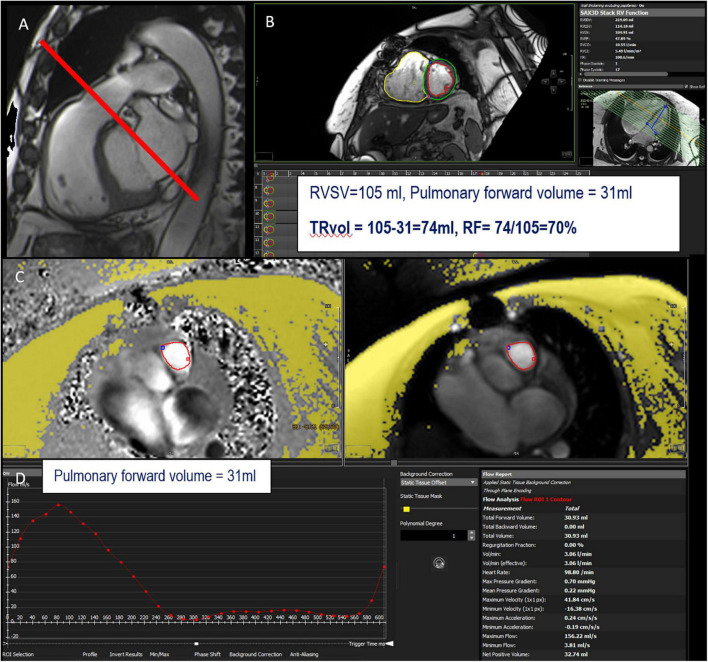
Example of TR assessment using indirect method n°1. RVOT bSSFP images showing slice position in the pulmonary artery (red line) for phase-contrast velocity mapping to obtain pulmonary forward volume **(A)**. Assessment of right ventricular stroke volume (RVSV) from short-axis cine SSFP images **(B)**. Phase and magnitude images with delineation of the pulmonary artery **(C)** allowing flow curves **(D)**. This patient has a severe TR with a tricuspid regurgitant volume (TRvol) of 74 ml and a regurgitant fraction (RF) of 70%.

Similar to MR, the regurgitant volume of TR (TRvol) can be measured indirectly as the difference between RVSV and PFF (or AFF in the absence of AR) using 2D cine (for RVSV) and retrospective acquisition with velocity-encoded phase-contrast sequence in a through–plane on the pulmonary valve (or the aortic valve), 5 mm above the valve (12) (Figure 7).

This is the most widely used CMR method and is valid in the absence of intra-ventricular shunts (79). The advantages and technical considerations are similar to those for MR and have already been discussed in the MR chapter.

#### Indirect Method n°2: Volumetric Method: 2D Cine Imaging

As for MR and AR, the volumetric method can be used to quantify the TRvol, expressed as the difference between RVSV and LVSV in the absence of other forms of valvular regurgitation. The advantages and technical considerations are similar to those for left-sided valvular regurgitation and have already been discussed in the MR and AR chapter.

These two indirect methods can be performed in addition to echocardiography to increase the confidence in the assessment of TR severity.

#### Direct Method: 2D Cine Phase-Contrast Velocity Mapping

2D PC imaging also allows the direct measurement of TR regurgitant flow through the tricuspid valve. As for MR, this technique is limited by significant motion and the saddle-shape of the tricuspid annulus and is not commonly used in clinical practice.

#### 4D Flow

Data concerning 4D flow in TR are very scarce. One recent study have suggested high concordance of 4D flow with standard 2D PC (indirect method) (80).

#### Late Gadolinium Enhancement Imaging and T1 Mapping

Unlike LV, tissue characterization (LGE and T1 mapping) of the RV is more complex due to the thin RV wall (thickness 1–3 mm), which creates partial volume effects. Transmural RV enhancement can be visualized, but non-transmural lesions are very challenging to visualize. LGE patterns may help to understand the underlying mechanism of TR, as it allows to detect ischemic patterns associated with pulmonary hypertension, or fibrosis associated with cardiomyopathies such as arrhythmogenic right ventricular cardiomyopathy. T1 assessment in TR has not yet been reported.

### Comparison of Cardiovascular Magnetic Resonance and Echocardiography on TRvol and Tricuspid Regurgitation Severity

Calculation of TRvol by echocardiography is more challenging than for MR and AR, and the current guidelines place CMR-derived TRvol as an alternative (4) (Table 4). Unlike left-sided regurgitant valvular lesions, quantitative assessment of TR by CMR is largely unexplored, and studies comparing these two imaging modalities are scarce (80, 81).

The largest and more recent study to compare echocardiography (integrative approach) and CMR (2D PC indirect method) has used the same thresholds defined in the ASE guidelines (36): TRvol mild: < 30 ml, moderate: 30–44 ml, and severe: ≥ 45 ml. Modest but significant correlations were found between quantitative echocardiographic measurements (vena contracta, effective regurgitant orifice area, and PISA-derived TRvol) and CMR (from 0.3 to 0.49) (81).

#### Cardiovascular Magnetic Resonance for Grading Severe Tricuspid Regurgitation and for the Timing of Surgery

As for MR and AR, using echocardiographic thresholds is inaccurate and extrapolating data from MR is too simplistic given the differences in anatomy, regurgitant orifice and shape, and hemodynamics between the two valves. A recent study by Zhan et al. proposed a different approach by defining optimal CMR thresholds for TRvol and RF for a low, intermediate, and high risk of mortality (81). The study by Zhan et al. has the merit of providing CMR thresholds for mortality risk, independent of the strong confounding effect of RV dilatation and dysfunction. Due to the lack of randomized studies using these CMR thresholds, the benefit of TR correction (surgery or percutaneous device) is still unknown. Current guidelines, mostly based on expert opinion, recommend intervention for patients with severe TR and concomitant left-sided valve surgery (class I) (4). In the setting of severe isolated TR, surgery is recommended for symptomatic patients without RV dysfunction (class I) or asymptomatic patients with RV dilatation or RV dysfunction (class IIa), without defined thresholds. However, CMR could contribute to defining these thresholds. Indeed, in a cohort of 76 patients undergoing tricuspid surgery, Park et al. found that CMR-based RVEF was an independent predictor for cardiac mortality and major post-operative cardiac events, with a cutoff of 46% (82). A recent observational prospective study by Rodriguez et al., on 43 patients with severe echocardiography-based TR undergoing tricuspid annuloplasty suggested that RVEDV indexed by CMR is the best independent predictor of overall mortality at follow-up, with a cut-off of 104 ml/m^2^, associated with higher cardiovascular mortality (83).

Aside from further pending CMR studies on specific thresholds, those defined by Zhan et al. (81) could be used in clinical practice and added to the established relevant value of CMR for RV volume and function.

## Pulmonary Regurgitation

Trace to mild pulmonary regurgitation (PR) is common in the general population, with little clinical significance. By contrast, significant PR is uncommon and usually related to either congenital heart disease (primary PR with abnormal leaflets) or pulmonary hypertension [secondary PR, normal pulmonary valve (PV) leaflets]. Echocardiography is the initial imaging modality to assess PR. However, the position of the valve behind the sternum, which makes visualizing the PV and RVOT difficult and thus the derived measurement for PR quantification, does not position echocardiography as the preferred method for PR assessment. Since CMR can provide unrestricted image planes for PV anatomy and RV function, it plays a crucial role in PR assessment, especially for patients with repaired tetralogy of Fallot (rTOF).

### Cardiovascular Magnetic Resonance for Assessing the Cause of Pulmonary Regurgitation

#### 2D Cine Imaging for Right Ventricle Volume and Pulmonary Valve Morphology

The assessment of RV structure and function is essential for determining the prognosis and planning surgical/percutaneous intervention for patients with rTOF (84).

**FIGURE 8:**
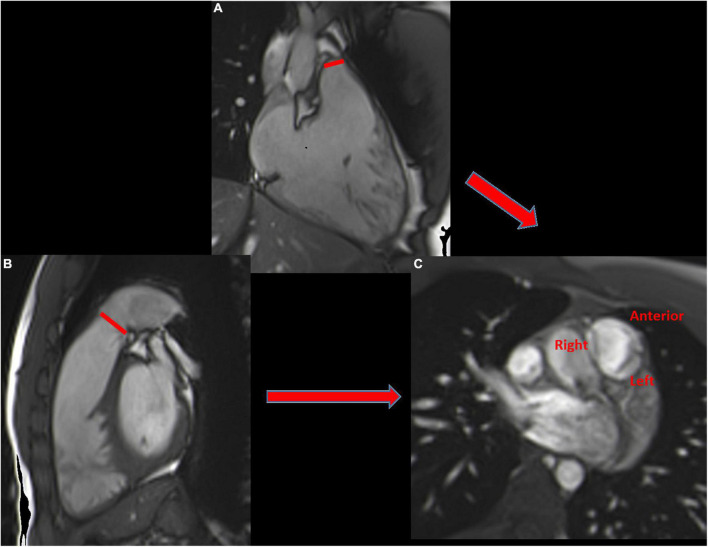
The 3-chamber view **(A)** and the RVOT **(B)** allowing the visualization of the three pulmonary leaflets (anterior, left and right) **(C)**.

Contrary to echocardiography, CMR provides good visualization of RVOT anatomy using a stack of SSFP cine images in the short and long axes (RVOT and 3-chamber). The anatomy of the pulmonary valve, with three semi lunar leaflets (anterior, left and right), can be visualized in through-plane images on the valve (Figure 8).

#### 2D Cine Imaging for Regurgitant Jet Visualization

PR can be seen as a dark jet in cases of flow turbulence in diastole, extending into the RVOT. However, in cases of laminar flow with a wide jet, there is almost no turbulence, making the jet nearly invisible. As for other forms of valvular regurgitation, it is a gross assessment and is not alone reliable.

### Cardiovascular Magnetic Resonance for Quantifying Pulmonary Regurgitation

#### Direct Method: Phase-Contrast Velocity Mapping

**FIGURE 9:**
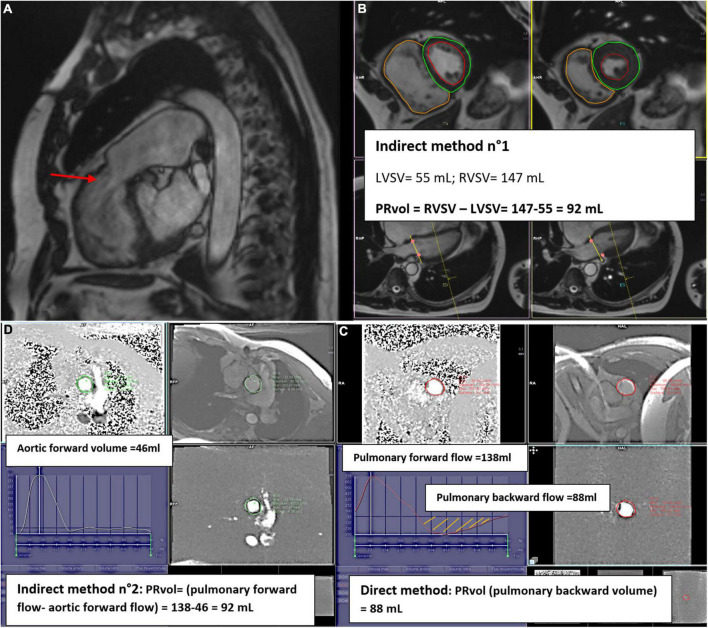
Cardiac magnetic resonance examination of a patient with severe pulmonary regurgitation (PR) using 3 different methods. RVOT view, showing flow void of PR (red arrow) **(A)**. PR quantification with the indirect method n°1 **(B)**: Assessment of left (LV) and right ventricular (RV) size and function by cine bSSFP; RV is severely enlarged, with a D shape (diastolic RV overload); PR Volume is the difference between RV and LV stroke volumes (SV). PR quantification with the direct method **(C)**: phase contrast flow mapping showing pulmonary forward volume at 138 mL and pulmonary backward volume of 88 mL [area under the diastolic flow curve (orange hatch lines)] corresponding to PRVol. Phase contrast flow mapping above the aortic valve, allowing aortic forward flow calculation at 46 mL. The difference between pulmonary and aortic forward flow gives PRvol (92 mL) **(D)**.

Similar to AR, direct measurement of pulmonary backward flow is feasible using 2D-PC with retrospective acquisition in a through-plane acquisition on the pulmonary valve, 5 mm to 1 cm above the level of the valve (12) (Figure 9).

This is the most widely used method and the current reference standard technique (85). In patients with rTOF or dilated pulmonary artery, pulmonary flow can be non-laminar and turbulent, leading to a possible underestimation of pulmonary regurgitant volume (PRvol). In this case, the volumetric method or 2D-PC acquisition perpendicular to the right and left pulmonary arteries can be performed to confirm the findings.

#### Indirect Method n°1: Volumetric Method: 2D Cine Imaging

As for other forms of valvular regurgitation, the volumetric method can be used to quantify PRvol by comparing RVSV and LVSV on bSSFP short-axis cine images (Figure 9), in the absence of other regurgitant disease or intra-cardiac shunt.

#### Indirect Method n°2: 2D Phase-Contrast Velocity Mapping

PRvol can be measured indirectly as the difference between PFF and AFF in the absence of tricuspid regurgitation (Figure 9).

#### Indirect Method n°3: 2D Cine Imaging and 2D Phase-Contrast Velocity Mapping

PRvol can be measured indirectly as the difference between RVSV and AFF in the absence of tricuspid regurgitation.

#### Late Gadolinium Enhancement Imaging and T1 Mapping

The presence of focal RV and LV enhancement in patients with rTOF is a common finding, mostly located at surgical sites of the RVOT, the patching of the ventricular defect, and in the inferior RV insertion (86). Such focal fibrosis can be a substrate for arrhythmias. The RV ECV could be associated with major adverse cardiovascular events (87). However, due to the thin walls of the RV, the proximity to the blood pool, and epicardial fat, RV ECV measurement is subject to potential errors.

### Comparison of Cardiovascular Magnetic Resonance and Echocardiography on PRvol and Pulmonary Regurgitation Severity

Most of the studies comparing echocardiography and CMR in PR assessment have been performed on patients with rTOF, with CMR as the gold standard (88–90).

#### Cardiovascular Magnetic Resonance for Grading Severe Pulmonary Regurgitation and for the Timing of Surgery

The CMR thresholds to define significant PR are the subject of debate. Several published studies have classified PR as significant if the RF ≥ 20% (91, 92) and others have used three grades of RF: mild < 20%, moderate: 20–40%, and severe > 40% (88, 89). Surprisingly, specific CMR PRvol thresholds have not been yet defined, despite a study showing better identification of severe RV dilatation using the PRvol than RF (92). Severe PR results in significant RV dilatation and eventually RV dysfunction, which can be irreversible, particularly for patients with rTOF. In this population, many centers recommend early PV intervention before symptoms or marked RV dilatation. CMR plays a crucial role in defining the optimal timing for PR intervention due to its ability to measure EF and RV dilatation with high accuracy. Geva et al. proposed a surgical approach for patients with a RF ≥ 25% and at least two of the following parameters: indexed RV end diastolic volume ≥ 160 ml/m^2^, indexed RV end systolic volume ≥ 70 ml/m^2^, indexed LV end diastolic volume ≤ 65 ml/m^2^ and RVEF ≤ 45% (84). Since the development of percutaneous valve procedures as an alternative to surgery, lower thresholds may be applied, although studies are lacking to provide guidance.

As for the other regurgitation, CMR is indicated in the case of inconclusive echocardiography (Table 4); however, CMR is the preferred method of non-invasive imaging to quantify PR, follow patients with rTOF, and guide the surgical intervention.

## Conclusion

Cardiovascular magnetic resonance has become a robust and reliable imaging modality, not only for the assessment of ventricle structure and function but also for the quantification of valvular heart regurgitation. Using comprehensive techniques, CMR allows an accurate measurement of valvular regurgitant volume (Rvol) and regurgitant fraction (RF) independent of jet morphology and direction. However, large studies are needed to validate the severity and prognosis of valvular heart regurgitation using CMR. Notwithstanding this, there is growing evidence showing that CMR could be an accurate complementary method to echocardiography for the measurement of Rvol and RF for grading regurgitation severity, particularly for mitral valve regurgitation (MR) and aortic valve regurgitation (AR). Emerging techniques, such as 4D flow are promising to enhance the accuracy of CMR for the quantification of valvular heart regurgitation. Furthermore, due to its unique ability to assess focal and diffuse LV fibrosis, CMR provides potential information in the clinical evaluation of MR and AR for planning and deciding the timing and indication of a specific therapy or intervention. Accordingly, taking into consideration the potential accurate uses of CMR in the evaluation of valvular regurgitation, this imaging modality could be useful in the assessment of valvular heart regurgitation in the setting of unclear severity of valvular regurgitation (potentially severe) measured by echocardiography.

## Author Contributions

EV provided conception and design of the research and wrote the manucript. EV, LI, FL, and CR provided iconography. BG, SM, YB, and CT provided critical revision of the manuscript. All authors contributed to manuscript revision, read and approved the submitted version.

## Conflict of Interest

The authors declare that the research was conducted in the absence of any commercial or financial relationships that could be construed as a potential conflict of interest.

## Publisher’s Note

All claims expressed in this article are solely those of the authors and do not necessarily represent those of their affiliated organizations, or those of the publisher, the editors and the reviewers. Any product that may be evaluated in this article, or claim that may be made by its manufacturer, is not guaranteed or endorsed by the publisher.
